# The Promise of Artificial Intelligence in Peyronie’s Disease

**DOI:** 10.1007/s11934-024-01233-5

**Published:** 2024-09-21

**Authors:** Thiago P. Furtado, Vadim Osadchiy, Sriram V. Eleswarapu

**Affiliations:** Division of Andrology, Department of Urology, David Geffen School of Medicine at UCLA, 10945 Le Conte Avenue, Suite #3361, Los Angeles, CA 90095 USA

**Keywords:** Peyronie’s disease, Artificial intelligence, 3d modeling, Decision-making, Penile curvature

## Abstract

**Purpose of Review:**

The application of artificial intelligence (AI) to enhance clinical decision-making in Peyronie’s disease (PD) has generated significant interest. This review explores the current landscape of AI in PD evaluation.

**Recent Findings:**

Recent advances in 3D modeling offer a more sophisticated approach to assessing PD deformities; however, the implementation of 3D modeling in clinical practice faces challenges, including the need for specialized equipment and time-consuming data processing, sometimes taking several hours of labor. AI holds promise for overcoming these hurdles through its ability to efficiently process large volumes of data and to perform accurate predictions based on such data.

**Summary:**

Future integration of AI with 3D modeling techniques could revolutionize PD evaluation by improving patient counseling, surgical planning, and clinical decision-making. Significant gaps in the literature have yet to be addressed, including the absence of robust evidence that incorporating such technology is superior to standard diagnostics.

## Introduction

Recently, there has been significant excitement over the application of artificial intelligence (AI) tools in healthcare [1]. The practical adoption of such AI tools outside of the investigational context has been largely absent. One area where AI has the potential to aid is in clinical decision-making related to Peyronie’s disease (PD).

PD is characterized by fibrotic plaque formation within the tunica albuginea, resulting in deformity, curvature, pain, and psychological distress [2]. Erect penile goniometry and penile duplex ultrasonography comprise the standard of care for PD diagnostics [3]. Unfortunately, these do not capture the heterogeneity inherent to PD, especially for men with more complex deformities associated with volume loss (e.g., hourglass deformity or indentation). However, even in the most straightforward cases, goniometry often lacks considerable inter-observer reliability. Ziegelmann et al.. have demonstrated differences as much as 20° in angle precision based on the drawing of virtual lines on the penis [3]. This variability has critical implications for clinical and research outcomes, directly influencing patient management decisions and insurance coverage for therapeutics that have only been approved for use within a certain range of deformity angulations, such as intralesional collagenase *Clostridium histolyticum*.

More sophisticated approaches to PD diagnostics have been explored. These involve mobile applications which attempt to standardize patients’ self-photography of their deformities, 3D photographic tools, and 3D modeling. 3D modeling offers the greatest potential to more accurately assess both curvature and volumetric changes. The implementation of 3D modeling in clinical practice, however, faces significant hurdles such as the need for specialized equipment and time-consuming data cleaning/organizing methodology, which may require, even in the most skilled hands, multiple hours for 3D image post-processing.

The ability of AI to accurately and efficiently process large volumes of data and make fairly accurate predictions based on such data holds great promise in advancing the state of PD diagnostics. Here, we will define commonly used terminology in the AI space and provide a comprehensive review of the literature on the use of AI and associated methodologies in PD assessment. Given the limited number of published studies on this topic, we have incorporated a small number of recent conference abstracts; these are obviously annotated as such in the text and the results of these data should be interpreted with a higher degree of trepidation. We will focus on the potential of AI to revolutionize clinical decision-making through improved accuracy and efficiency in 3D modeling.

## Key Definitions

**AI** can be difficult to define, as it is often context dependent and encompasses not just computer science, but also other related fields such as psychology, philosophy, and linguistics, among others [4, 5]. Broadly speaking, it can be thought of as the branch of science that attempts to use computers to perform tasks that would otherwise require human-level oversight, attention, and intelligence. Some have defined AI as the science of making “intelligent machines.” The below defined terms of machine learning, neural networks, and deep learning fall under the purview of AI. In the medical literature, these terms are sometimes erroneously used interchangeably.

**Machine learning** describes the process by which a dynamic algorithm allows a computer to learn and identify patterns based on a training set of data [5]. This new “knowledge” can then be applied to a test set of data to make predictions. The benefit of such a system allows for the relatively rapid consolidation of data in ways that may not be immediately apparent to a human. The quality of the model is dependent on the quality of the training set initially provided [4]. An example of this would be the development of a machine learning classifier that can distinguish between overweight versus obese individuals with greater than 90% accuracy based solely on stool metabolite and neuroimaging data; this classifier was trained on over three thousand distinct clinical datapoints but ultimately identified only 126 data points as meaningful for classification [6]. 

**Artificial neural networks**, sometimes shortened simply to neural networks, gets their name from the networks identified in the central nervous systems of animals [5]. In general, a neural network consists of nodes connected to other nodes with a certain strength. The output of one node represents the input of the next. These nodes are arranged in an interconnected network; the strengths or weights of these inter-nodal connections can be adjusted based on the inputs received from other nodes [4, 5]. As new data is introduced, weights are adjusted to strengthen certain neural pathways, while weakening others. Such methodology has been applied, for example, to query the effects of vitamin D supplementation on the development of breast cancer in a high-risk cohort of women. For this model, the neural network was provided clinical parameters in addition to baseline and follow-up mammograms [7]. 

**Deep learning** describes a type of machine learning methodology that uses multiple layers of neural networks to identify and predict patterns even in unstructured data [5]. This technology can analyze data in media (video, audio, etc.) to recognize and extract important pieces of information, and to use this data to perform complex tasks without human intervention [4]. Deep learning has previously been explored within the context of assigning Gleason scores to prostate specimens to assist pathologists in streamlining certain diagnostic workflows [8]. 

## 3D Modeling in PD Evaluation

Three-dimensional modeling has emerged as a potentially valuable tool in the evaluation and management of PD and penile curvature, offering enhanced visualization of penile deformities that traditional erect penile goniometry and 2D photography lack. PD curvature often involves multiple planes and can be associated with volumetric changes such as indentations and hour-glassing. These cases cannot be captured adequately with goniometry or 2D photography alone. Evaluation can be further complicated by the presence of multiple PD plaques causing complex deformities. A small group of published studies have described the feasibility and reliability of 3D modeling; of note, the methodology for these studies varies widely from the equipment used, to the actual set-up, and the software for modeling.

Margolin et al.. (2017) described a proof-of-concept methodology leveraging 3D photogrammetry in which the investigators were able to capture full-color, 3D models from a series of 2D photographs using an infrared light camera device attached to an iPad [9]. They applied this technique to printed penile models, which were reconstructed digitally and analyzed using a 3D computer graphics software to compute volume, circumference, and angle of curvature. They report accurate and reliable measurements for volume-loss deformities, though they do overestimate erect penile volume and underestimate erect penile volume loss by an average of 7.1% and 1.9%, respectively. Inter-observer and inter-test correlations were rated as good, with correlation coefficients over 0.75. The study also demonstrated a photograph acquisition time of less than one minute and under five minutes to reconstruct and analyze the images. The same group presented a follow-up pilot study as an abstract one year later with 14 participants; however, this has not yet gone through comprehensive peer-review.

Siapno et al.. (2020) tested the accuracy of a 3D structured light scanner device (Artec Space Spider) for angulation measurements on printed blocks, comparing it to goniometry, traditional photography, and 3D photography [10]. Unlike 3D photography, which utilizes many 2D pictures to compose a 3D model of an object, structured light scanning maps the contours of the object by emitting light and capturing how the model reflects the source. Their study demonstrated high accuracy, precision, and reliability of handheld 3D mapping technologies in a laboratory setting (Interclass Correlation Coefficient: 0.99%; 95% CI 0.999, 0.999). The same group published a proof-of-concept study in 2021 testing the same structured light scanner device, but in 3D-printed penile phantoms with angulation deformities [11]. They reported remarkable accuracy in the measurements compared to the digital phantom standard. These results have not been validated within a clinical setting, although the authors are enrolling patient volunteers to test the methodology alongside standard-of-care evaluation techniques.

In 2021, Pavone et al.. conducted a pilot study to assess the feasibility and clinical utility of using 3D reconstruction for the evaluation of penile curvature in patients with PD [12]. Utilizing a sequence of up to 50 photographs taken with a smartphone after a pharmacologically induced erection, the images were processed with 3DF Zephyr, MeshLab, and Blender software to create a detailed 3D model of the penile curvature. This model was used for preoperative counseling and surgical planning. The study included four patients, with a median photograph acquisition time of 39 s and a processing time of nearly six hours. Both patients and surgeons rated the method highly, finding it useful for understanding the patient’s specific pathology and for surgical planning. The lengthy processing time length may limit the application of this technology in clinical use.

An abstract by Thorogood et al.. (2024) explored the application of 3D modeling in clinical assessment of penile curvature for PD [13]. Using the MirrorMe3D platform, two trained urology fellows created models from photographs taken with a smartphone and processed them to produce 3D images. The study generated high-quality 3D models from seven patients, with image acquisition taking under two minutes and curvature assessment an additional five minutes. High agreement was observed between coders on curvature measurements within a 10° margin. Despite success with this methodology, the authors highlight important limitations involved in the application of this technology to a clinical setting including inconsistent lighting conditions and patient anxiety. Similar findings were obtained by another abstract published by Nascimento et al.. (2023) but by leveraging intraoperative video images [14]. The scientific rigor of this work has yet to be adequately assessed as these projects have not yet gone through full peer-review.

Despite the advantages, 3D modeling in PD evaluation faces several challenges. The highest quality and perhaps most robust data for the application of 3D modeling in PD comes from production of models based on 3D printed phantoms taken under ideal laboratory settings. Although some feasibility of such technology in a clinical environment has been demonstrated in pilot studies, the bulk of these studies have only been published as conference abstracts and have not yet gone through the peer-review process. Furthermore, the challenges of implementing this technology for patients with PD are myriad, including both logistical concerns (e.g., setting up 3D imaging equipment in a dedicated space, training staff, defining workflows) and technical considerations (e.g., ensuring consistent lighting, proper angles). Moreover, processing the images to create accurate 3D models can take several minutes to several hours, depending on the software and computational resources available​​. The current state of the literature in this realm is highly susceptible to publication bias; furthermore, studies that have been published might omit or understate the burden and complexity of 3D modeling to enhance positive outcomes.

## AI for Penile Curvature Assessment

AI has shown promise in automating the assessment of penile curvature, particularly in the hypospadias literature, where accurate measurement is critical for surgical decision-making.

Baray et al.. (2023) developed an automated deep learning-based method for accurately measuring penile curvature using 2D images [15]. Researchers used a set of nine 3D-printed models to generate 913 images of penile curvature ranging from 18° to 86°. The penile region was localized and cropped using a single-stage object detector model, followed by shaft extraction using neural network models. Four distinct points on the shaft were identified to measure curvature, and a neural network model (HRNet) was trained to predict these landmarks and calculate the curvature angle. The optimized HRNet model was then applied to four medical images of real patients retrieved from publicly available sources. The method achieved a mean absolute error (MAE) of less than 5° for both model images and their derivative masks. For real patient images, AI predictions varied between 1.7° and 6° compared with clinical expert assessments. This novel approach demonstrates a reliable method for the automated measurement of penile curvature, potentially improving clinical assessments by surgeons and researchers.

Abbas et al. (2022) developed an AI-based method for automated measurement of penile curvature using 2D images, leveraging a combination of object detection AI for penile area localization, a neural network-based model for shaft segmentation, and a custom digital image processing algorithm for angle estimation [16]. The study utilized a dataset of 900 images of 3D-printed penile models with curvature angles ranging from 18° to 88°. The framework demonstrated robust performance with a mean average precision of 99.4% for penile area detection. Curvature angle estimation achieved a MAE of 8.5°. This approach could significantly improve the precision of penile curvature measurements by reducing inter-surgeon variability, offering a reliable tool for clinical assessments.

This same group also conducted a study to validate an AI-based tool for automated penile curvature estimation, employing a novel quantification method [17]. Seven 3D-printed penile models with various curvature angles ranging from 33° to 88° were used to assess and compare the interobserver agreement of different penile curvature measurement techniques, including visual inspection, goniometry, manual estimation via a mobile application, and the AI-based tool. Thirty-five pediatric urology practitioners participated, providing data on the time required, MAE, and inter-rater agreement for each method. Results indicated that the AI-based tool achieved the lowest MAE and highest inter-class correlation, with an average measurement time of just 22 s. The AI tool also demonstrated superior accuracy and consistency compared to traditional methods, making it a promising option for clinical use in penile curvature assessment, particularly for mild and moderate cases.

## Perspective for Future Integration of AI and 3D Modeling

Three-dimensional modeling has been shown to be a potential solution for the evaluation of penile deformities, especially in PD where volume-loss lesions are commonly found, yet cannot be accurately accounted for with conventional clinical tools. Many proposed techniques share the limitation of long processing times, which may make implementation challenging in a busy clinical environment. AI-based tools show significant promise in improving efficiency and addressing many of the pitfalls of in-practice 3D modeling implementation. Although we, and along with many other groups, hypothesize that the incorporation of 3D modeling and AI into each phase of PD care (initial encounter, pre-intervention, post-intervention follow-up) will improve clinical decision-making and patient-centered outcomes including expectation management, this question has not yet been rigorously studied in the available literature.

Most of the published techniques are designed as proof-of-concept studies, and there is low evidence of their feasibility in real practice. There is also a notable absence of studies that feature the application of such technology on a larger volume (> 20) of patients, which perhaps speaks to the difficulties in translating this methodology into clinical practice.

## Conclusions

The integration of AI and advanced 3D modeling holds significant potential for improving the evaluation and management of PD. Future developments that leverage AI may enhance image quality, automate measurements, and streamline clinical workflows, ultimately improving penile deformities evaluation, patient counseling, surgical planning, and clinical decision making.

## Key References

- Walker DT, Jiang T, Santamaria A, Osadchiy V, Daniels D, Sturm RM, et al. 3D-printed phantoms to quantify accuracy and variability of goniometric and volumetric assessment of Peyronie’s disease deformities. Int J Impot Res. 2022;34(8):786-9.
-
  - First study to assess a 3D structured light scanner device for PD’s 3D modeling.
- Pavone C, Abrate A, Altomare S, Vella M, Serretta V, Simonato A, et al. Is Kelami’s Method Still Useful in the Smartphone Era? The Virtual 3-Dimensional Reconstruction of Penile Curvature in Patients With Peyronie’s Disease: A Pilot Study. The Journal of Sexual Medicine. 2021;18(1):209 − 14.
-
  - Relevant study that utilizes smartphone photography to create detailed 3D models to evaluate penile curvature in patients with PD.
- Baray SB, Abdelmoniem M, Mahmud S, Kabir S, Faisal MAA, Chowdhury MEH, et al. Automated measurement of penile curvature using deep learning-based novel quantification method. Front Pediatr. 2023;11:1149318.
-
  - Relevant study showcasing the development and validation of an automated deep learning-based method for penile curvature measurement from 2D images.
- Abbas TO, AbdelMoniem M, Chowdhury MEH. Automated quantification of penile curvature using artificial intelligence. Front Artif Intell. 2022;5:954497.
-
  - Relevant study demonstrating the use of AI for penile curvature assessment and angle estimation.
- Abbas TO, AbdelMoniem M, Villanueva C, Al Hamidi Y, Elkadhi A, AlSalihi M, et al. Urologist validation of an artificial intelligence-based tool for automated estimation of penile curvature. J Pediatr Urol. 2024;20(1):90.e1-.e6.
-
  - Relevant study comparing a novel AI-based method for automated penile curvature estimation to traditional methods.

## Data Availability

No datasets were generated or analysed during the current study.
